# Role of Mineralocorticoid and Angiotensin Type 1 Receptors in the Paraventricular Nucleus in Angiotensin-Induced Hypertension

**DOI:** 10.3389/fphys.2021.640373

**Published:** 2021-03-08

**Authors:** Sandra L. Burke, Benjamin Barzel, Kristy L. Jackson, Cindy Gueguen, Morag J. Young, Geoffrey A. Head

**Affiliations:** ^1^Neuropharmacology Laboratory, Baker Heart and Diabetes Institute, Melbourne, VIC, Australia; ^2^Cardiovascular Endocrinology Laboratory, Baker Heart and Diabetes Institute, Melbourne, VIC, Australia; ^3^Department of Pharmacology, Monash University, Clayton, VIC, Australia

**Keywords:** hypertension, sympathetic nerve activity, angiotensin II, aldosterone, RU28318, rabbit

## Abstract

The hypothalamic paraventricular nucleus (PVN) is an important site where an interaction between circulating angiotensin (Ang) and mineralocorticoid receptor (MR) activity may modify sympathetic nerve activity (SNA) to influence long-term elevation of blood pressure. We examined in conscious Ang II-treated rabbits, the effects on blood pressure and tonic and reflex renal SNA (RSNA) of microinjecting into the PVN either RU28318 to block MR, losartan to block Ang (AT_1_) receptors or muscimol to inhibit GABA_*A*_ receptor agonist actions. Male rabbits received a moderate dose of Ang II (24 ng/kg/min subcutaneously) for 3 months (*n* = 13) or sham treatment (*n* = 13). At 3 months, blood pressure increased by +19% in the Ang II group compared to 10% in the sham (*P* = 0.022) but RSNA was similar. RU28318 lowered blood pressure in both Ang II and shams but had a greater effect on RSNA and heart rate in the Ang II-treated group (*P* < 0.05). Losartan also lowered RSNA, while muscimol produced sympatho-excitation in both groups. In Ang II-treated rabbits, RU28318 attenuated the blood pressure increase following chemoreceptor stimulation but did not affect responses to air jet stress. In contrast losartan and muscimol reduced blood pressure and RSNA responses to both hypoxia and air jet. While neither RU28318 nor losartan changed the RSNA baroreflex, RU28318 augmented the range of the heart rate baroreflex by 10% in Ang II-treated rabbits. Muscimol, however, augmented the RSNA baroreflex by 11% in sham animals and none of the treatments altered baroreflex sensitivity. In conclusion, 3 months of moderate Ang II treatment promotes activation of reflex RSNA principally via MR activation in the PVN, rather than via activation of AT_1_ receptors. However, the onset of hypertension is independent of both. Interestingly, the sympatho-excitatory effects of muscimol in both groups suggest that overall, the PVN regulates a tonic sympatho-inhibitory influence on blood pressure control.

## Introduction

The important contribution of an activated sympathetic nervous system (SNS) to hypertension is well established (Esler, 1995, 2015). However, a clear demonstration of the neurogenic contribution to angiotensin (Ang) dependent hypertension has been complicated by the direct vasoconstrictor actions of systemically administered Ang II on vascular and cardiac structure which influence neuroeffector function, and by chronic baroreflex suppression of the SNS (Moretti et al., 2009). To avoid the constrictor complications, an alternative approach is to use a low systemic dose of Ang II that does not elicit an immediate pressor response but that increases blood pressure in the long term in part via a centrally mediated increase in neurogenic vasomotor tone (Cox and Bishop, 1991). We developed a model of low level activation of the renin-angiotensin-aldosterone system (RAAS) in rabbits which involves a 12 week systemic infusion of a very low dose of Ang II producing only a modest increase in blood pressure but a substantial increase in renal sympathetic activity (RSNA; Moretti et al., 2012).

The brain regions first activated by low dose Ang II, demonstrated using Fos related antigen, are the circumventricular organs such as the subfornical organ (SFO) and organum vasculosum lamina terminalis (OVLT). This is due to the lack of blood brain barrier and high density of AT_1_ receptors (AT_1_R) in this region. However, the initial activity diminishes over time, possibly due to down-regulation of the AT_1_R (Davern and Head, 2007). Our data further show that four brain regions remain activated after 12 weeks of Ang II infusion: the OVLT, median preoptic nucleus, supraoptic nucleus (SON), and paraventricular nucleus (PVN). However, only the PVN showed a sustained activation of Fos that paralleled the sustained sympathetic activation (Moretti et al., 2012) and may therefore be the region driving the sympatho-excitation in the long term. Indeed, there is considerable evidence that circulating Ang II activates AT_1_R in the SFO which project to the PVN, inducing reactive oxygen species and increased expression of p47^*phox*^ and gp91^*phox*^ (Oliveira-Sales et al., 2009). Ang II may also directly activate AT_1_R in the PVN due to a breakdown of the blood brain barrier in subjects with hypertension. PVN neurons project to pre-sympathetic neurons in the spinal cord (Saper et al., 1976) and are prime candidates to mediate the sympatho-excitation observed with Ang II hypertension (Biancardi et al., 2014). This view is supported by studies showing knock down of AT_1_R only in the PVN prevents most of the increase in blood pressure (Chen A. et al., 2014).

There is evidence that in addition to AT_1_R activation in the PVN, mineralocorticoid receptors (MR) in this region may also be involved in promoting Ang II-induced hypertension. Elevated Ang II levels by exogenous infusion or stimulation of renin increase serum aldosterone levels through modulation of adrenal production. MR activation by aldosterone is central for sodium and water homeostasis in the kidney epithelial cells, but MR are expressed in many non-epithelial cell types including the brain (Oki et al., 2012; Chen J. et al., 2014; Joels and de Kloet, 2017). Of note, the MR are only activated by aldosterone in a limited number of tissues. This is due to the intrinsic equivalent affinity of the MR for both aldosterone and cortisol. Co-expression of a hydroxy steroid dehydrogenase type II enzyme (11bHSD2) metabolizes cortisol and prevents its access to the MR; this is well described in renal epithelial cells for the control of sodium and potassium homeostasis (Krozowski et al., 1995). MR that are not colocalized with 11bHSD2 respond to cortisol under normal physiological conditions, particularly in the brain (Iqbal et al., 2014; Joels and de Kloet, 2017). Moreover, in peripheral cells in the presence of cell stress the MR responds to cortisol as an agonist equivalent to aldosterone, suggesting a range of signaling outcomes for cortisol-bound MR (Ward et al., 2001).

While 11bHSD2 is widely expressed in the developing brain, it is only found in a small number of regions in the adult brain including the nucleus tractus solatarii (NTS) and the PVN. These regions are proposed to respond to aldosterone for the control of salt appetite and thirst (Geerling and Loewy, 2007; Oki et al., 2012; Chen J. et al., 2014; Haque et al., 2015). Intracerebroventricular (ICV) infusion of the MR antagonist RU28318 can reduce the blood pressure rise to systemic Ang II and other pressor agents such as aldosterone (Xue et al., 2011). Further, central infusion of an aldosterone synthase inhibitor (FAD286) or MR antagonist (spironolactone) prevented most of the Ang II-induced neuronal activation in the PVN and importantly the increase in blood pressure (Huang et al., 2010). By contrast, Fos in the SON was not altered, confirming the importance of the MR and potentially aldosterone in the PVN rather than the SON for modifying Ang II-induced hypertension. The question therefore is whether AT_1_R signaling with or without combined MR/aldosterone signaling in the PVN mediates the sympatho-excitation during low Ang II-mediated hypertension. To the best of our knowledge, no previous study has injected the antagonists directly into the PVN. Thus, the aims of the present study were to determine, in conscious rabbits (1) whether low level activation of the RAAS modeled by infusion of a low dose of Ang II for 12 weeks causes sustained activation of neurons in the PVN resulting in amplified sympathetic and pressor responses to stress, chemoreceptor activation and ultimately hypertension and (2) whether the process involves increased MR and/or AT_1_R activation in the PVN. Our approach was to deliver antagonists directly into the PVN in conscious rabbits and to test sympathetic responses to stimulation of chemoreceptor and stress pathways and to also examine the baroreflex. We blocked MR using the highly specific antagonist RU28318 (Young et al., 1995) and AT_1_R using losartan (Mayorov and Head, 2003). To determine the overall contribution of the PVN, we examined the effect of muscimol (Maiorov et al., 2000) as it provides neuronal inhibition via activation of GABA_*A*_ receptors.

## Materials and Methods

### Animals

Experiments were conducted in 26 male New Zealand White rabbits (initial body weight 2.3–3.1 kg). This study was approved by the Alfred Medical Research Education Precinct Animal Ethics Committee and conducted in accordance with the Australian Code of Practice for Scientific Use of Animals.

### Surgical Procedures and Protocol

**FIGURE 1:**
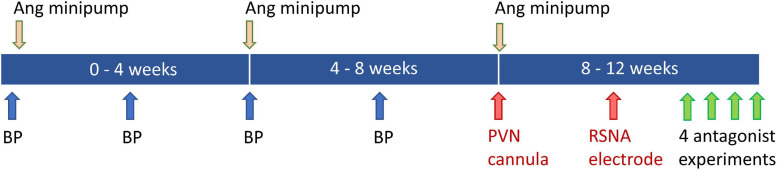
Schematic diagram showing timeline of blood pressure (BP) measurements at baseline and weeks 2, 4, and 6. Angiotensin (Ang) minipumps were inserted at baseline and changed at week 4 and 8. Surgery to implant the PVN cannula and renal sympathetic nerve activity (RSNA) electrode was carried out at weeks 8 and 10. Experiments to examine effects of antagonists, agonist and vehicle occurred during week 12.

Rabbits were randomly allocated to sham (*n* = 13) and Ang II (*n* = 13) groups. Experiments to measure mean arterial pressure (MAP) and heart rate over 1h were conducted at baseline and at 2, 4, and 6 weeks after beginning Ang II or sham treatment (Figure 1). Ang II (Auspep, Tullamarine, VIC, Australia), in a vehicle of 0.9% NaCl, was delivered by three sequential 28-day osmotic minipumps (Alzet Model 2ML4; Durect Corp., Cuertino, CA, United States) implanted subcutaneously under local anesthesia and sham-treated rabbits received a dummy minipump (Denton et al., 2001; Figure 1). The initial dose of Ang II was 20 ng/kg/min delivered at a rate of 2.5 μL/h. We aimed to increase MAP by 10–30% and if MAP was outside this range, the dose of Ang II was adjusted (Moretti et al., 2012). The average dose over 12 weeks was 24 ± 1 ng/kg/min.

At 8 and 10 weeks after the start of the Ang II infusion, rabbits underwent two surgical operations under isoflurane anesthesia (3–4% in 1L/min oxygen) following induction with propofol (10 mg/kg intravenous (i.v.), Fresenius Kabi, Pymble, NSW, Australia). Carprofen (3 mg/kg s.c., Pfizer, North Ryde, NSW, Australia) was given 30 min beforehand and 24 h later for analgesia. In the first operation, a bilateral brain cannula (22-gauge, Plastics One, Roanoke, VA, United States) was fitted above the PVN (nucleus coordinates from bregma −2.0 mm caudal, ±0.9 mm lateral to midline, and depth of 13.4 mm from the skull) (De Matteo et al., 2006a). The guide cannula was situated 2 mm above the PVN, ensuring that the nucleus was intact before experimentation (Lim et al., 2016). The minipump was also replaced at this time. In the second operation, an electrode was implanted on the left renal nerve for recording RSNA 1 week prior to the first experiment (Dorward et al., 1985).

### Experimental Procedures and Protocol

Experiments were conducted in conscious rabbits held in a standard single rabbit holding box. The central ear artery was catheterized with a 22G catheter (BD Insyte, Singapore) for arterial pressure and heart rate, derived from the pressure pulse. For the main experiments, a lateral ear vein was also catheterized for i.v. injections. The RSNA signal was recorded at bandwidth 50–1,000 Hz and was rectified and integrated (20 ms) with threshold adjusted to minimize noise. Burst amplitude and frequency were also measured (Lim et al., 2015).

A maximum of four drugs were administered to each rabbit in random order and on separate days with at least one-day recovery in between (minimum 48 h between injections). Baseline parameters were recorded for 1 h followed by bilateral administration of one of the drugs into the PVN using an injector 2 mm longer than the guide cannula (28-gauge, Plastics One) (Lim et al., 2016). One dose of either RU28318 (0.34 nmol equivalent to 150 ng in a volume of 400 nL, Tocris, Ellisville, MO, United States), losartan (5 nmol equivalent to 2.1 μg in 200 nL, Sigma-Aldrich Pty., Ltd., Sydney, Australia), muscimol (2 nmol in 200 nL, Sigma-Aldrich), or vehicle (200 nL Ringer’s solution, Baxter, Old Toongabbie, NSW, Australia) was administered over 30–60 s. Doses were based on previous experiments (Young et al., 1995; Ito and Sved, 1996; Maiorov et al., 2000; Mayorov and Head, 2003; De Matteo et al., 2006a, b). RU28318 at 10 ng is effective for 24 h after ICV injection in rats (Rahmouni et al., 2002). We have shown that 2 nmol losartan (half the dose we used in this study) injected into the rostroventral medulla lasts at least 2 h (Mayorov and Head, 2003). Sixty minutes after RU28318 and 30 min after the other drugs, rabbits were subjected to air jet stress (60 L/min for 10 min) and after 20-min recovery, hypoxia (10% O_2_ + 3% CO_2_ in N_2_) for 20 min. Both air jet stress and hypoxia produce marked sympathetic activation and provide a measure of the effects of the antagonists at high sympathetic activity (Moretti et al., 2012). Duplicate RSNA and heart rate baroreflexes were derived by i.v. infusions of 0.5 mg/mL phenylephrine (25 μg/kg) and 1 mg/mL sodium nitroprusside (30 μg/kg), respectively (Burke et al., 2016).

### Left Ventricular Weight and Confirmation of Cannula Placement

**FIGURE 2:**
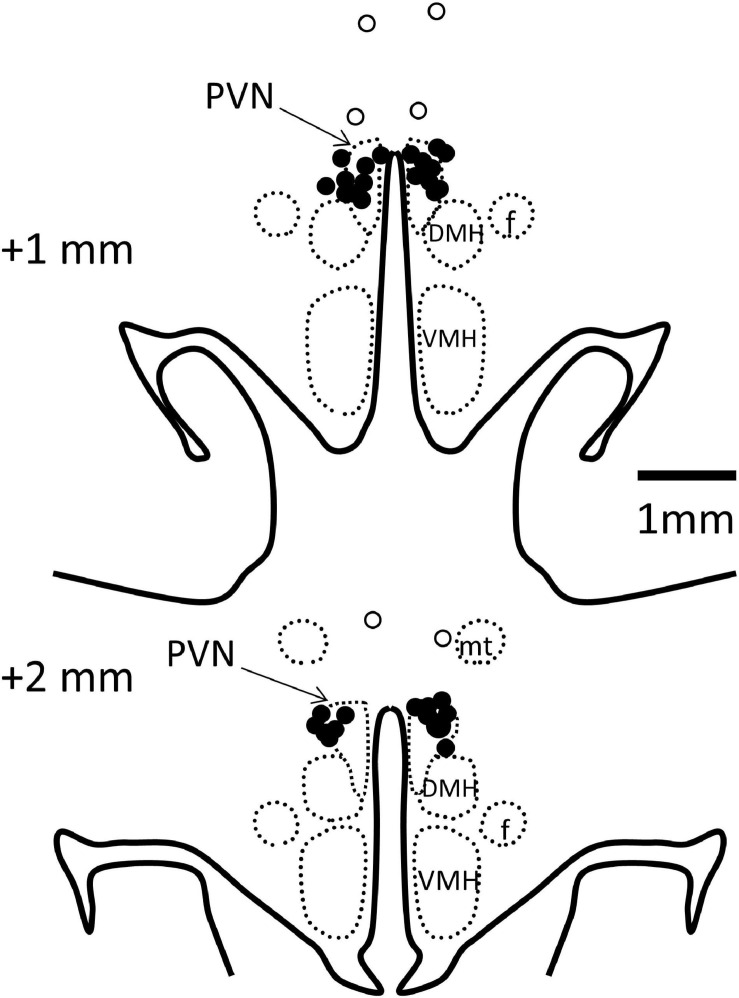
Schematic representation of microinjection sites. Coronal sections at 1 and 2 mm posterior to bregma with bilateral microinjection sites shown as filled circles when in the paraventricular nucleus (PVN, indicated by arrows, *n* = 19) and open circles when outside (*n* = 3). Injection sites in 1 other rabbit were outside the PVN, not visible on these sections. DMH, dorsomedial hypothalamus; VMH, ventromedial hypothalamus; f, fornix; mt, mammillothalamic tract.

Rabbits were killed by an anesthetic overdose (160 mg/kg i.v., sodium pentobarbitone, Virbac, Milperra, NSW, Australia) and the left ventricle weighed. PVN cannula placements were verified by injection of methylene blue dye before perfusion fixation of the brain and sectioning (40 μm) in a cryostat. In 19 rabbits, microinjection sites were confirmed in the PVN (Figure 2). Four rabbits with injection sites outside the PVN were excluded from the study as were three rabbits which did not make a full recovery from PVN cannula surgery and one in which the MAP did not meet the criterion of an increase of 10–30%.

### Data Analysis

Mean arterial pressure, heart rate, and RSNA were digitized at 1,000 Hz using an analog-to-digital data acquisition card (National Instruments 6024E, Austin, TX, United States) and averaged over 2 s. To allow for between animal comparisons, RSNA was normalized to the maximum RSNA value elicited by the smoke-induced nasopharyngeal reflex (100 normalized units, nu) (Burke et al., 2016). Baseline data were averaged over 1 h at the beginning of each experiment. MAP–RSNA and MAP–heart rate baroreflex curves were fitted to a sigmoidal curve (Ricketts and Head, 1999). The responses to air jet stress were averaged over the last 9 min and those to hypoxia over the last 10 min of exposure (Lim et al., 2015).

### Statistical Analysis

Values are expressed as mean ± SEM or mean difference ± SE of the difference (SED) from control. Data were analyzed by split plot repeated measures (mixed model) analysis of variance (ANOVA). The between-groups sums of squares were partitioned into the effect of drug compared to control within groups (P_*control*_) and the effects of Ang II compared to sham (P_*sham*_) or antagonist/agonist compared to vehicle (P_*veh*_). One-way analysis of variance was used for data collected at a single time point. Type 1 error was controlled using Bonferroni adjustment to the probability threshold and Greenhouse-Geisser correction to account for non-sphericity (reduce inflated residual degrees of freedom). A probability of *P* < 0.05 was considered significant.

## Results

### Effect of Ang II on Cardiovascular Variables, RSNA, Body Weight and Left Ventricular Weight

**FIGURE 3:**
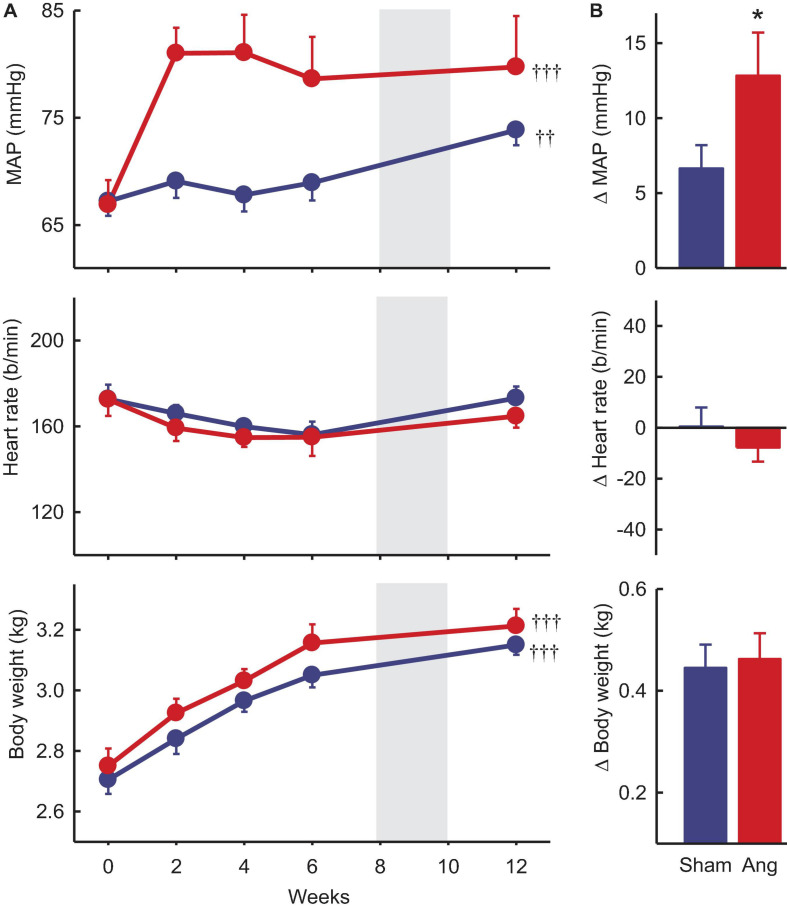
Mean arterial pressure (MAP), heart rate and body weight at baseline and after Ang II or sham treatment. **(A)** Average values at baseline (week 0) and to 12 weeks after beginning Ang II (red, *n* = 8) and sham (blue, *n* = 10) treatment. Data are mean ± SEM. Gray panel shows the period when surgery occurred. ^††^*P* < 0.05 and ^†††^*P* < 0.001 for week 12 vs week 0. **(B)** Average changes from baseline at week 12. Data are mean difference ± SE of the difference. *P < 0.05 for Ang II vs sham.

Baseline MAP and heart rate were 67.1 ± 1.2 mmHg and 173 ± 5b/min, respectively, averaged over all rabbits included in the study. In sham rabbits with a dummy minipump, MAP remained stable until after the period of surgery at 8–10 weeks when there was a small rise in MAP (+6.7 ± 1.4mmHg, +10%, P_*baseline*_ = 0.019). Heart rate at week 12 was similar to baseline (Figure 3).

Two weeks after starting Ang II treatment, the average increase in MAP from baseline was 14.1 ± 2.8 mmHg (+22%, *n* = 8, P_*baseline*_ < 0.001) and MAP remained elevated until week 12 (79.7 ± 4.8 mmHg, +19%, P_*sham*_ < 0.05, Figure 3). At this time, average MAP was 5.9 mmHg less in sham rabbits (P_*sham*_ = 0.022, Table 1). In Ang II rabbits there was a steady decline in heart rate but at week 12, one week after surgery, there was a small reversal in the trend and heart rate was similar to baseline and to the sham group (*P*_*sham*_ = 0.063, Figure 3, and Table 1).

**TABLE 1:** Cardiovascular parameters and renal sympathetic nerve activity (RSNA) after 12 weeks Ang II or sham treatment.

	Sham	Ang II	P_*sham*_
MAP (mmHg)	73.9 ± 1.4	79.7 ± 4.8	**0.022**
Heart rate (b/min)	173 ± 5	165 ± 5	0.063
Total RSNA (nu)	7.4 ± 1.3	7.2 ± 0.3	>0.5
Total RSNA (μV)	30.0 ± 5.0	26.3 ± 5.0	0.36
Burst Amplitude (nu)	29.0 ± 3.9	31.0 ± 2.2	>0.5
Burst Amplitude (μV)	129 ± 20	132 ± 25	>0.5
Frequency (bursts/s)	5.0 ± 0.4	5.4 ± 0.4	0.29
Nasopharyngeal (μV)	596 ± 102	573 ± 168	>0.5
n	10	8	

*One-hour control data averaged over 4 experiments at week 12. Data are mean ± SEM. P_*sham*_ for Ang II vs sham, shown in bold if *P* < 0.05.*

At week 12, normalized RSNA was similar in sham and Ang II groups (7.4 ± 1.3nu and 7.2 ± 0.3nu, *P* > 0.5, Table 1) and this was also the case when RSNA was expressed in microvolts, as burst amplitude or burst frequency (Table 1).

Average baseline body weight was 2.66 ± 0.04 kg in all rabbits. In sham and Ang II rabbits, body weight increased similarly (+0.45 ± 0.05 kg and +0.46 ± 0.05 kg, +17%, P_*sham*_ = 0.70, Figure 3) over the 12 weeks.

Left ventricular weights relative to body weight were 20% greater in the Ang II compared to sham group (1.60 ± 0.07 g/kg vs 1.33 ± 0.04 g/kg, P_*sham*_ = 0.003).

### Effect of the Antagonists and Agonist on Cardiovascular Variables and Tonic RSNA

**FIGURE 4:**
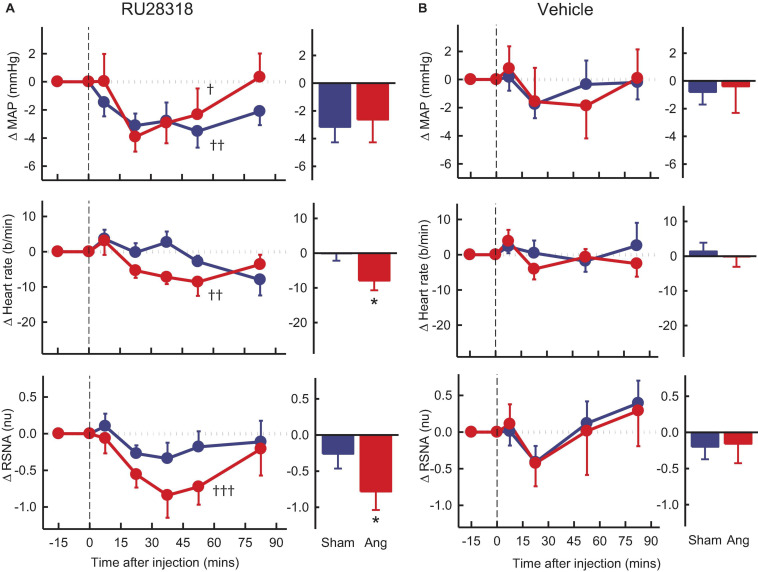
Average changes in mean arterial pressure (MAP), heart rate and renal sympathetic nerve activity (RSNA, normalized units) in rabbits after bilateral injection into the PVN of RU28318 and vehicle. **(A)** Line graphs are mean difference from control ± SED after RU28318 (0.34 nmol) injection shown at dashed vertical line in sham (blue circles, *n* = 10) and Ang II (red circles, *n* = 8) rabbits, ^†^*P* < 0.05, ^††^*P* < 0.01, and ^†††^*P* < 0.001 for change from control at 30–60 min. Bar graphs show average difference from control ± SED at 30–60 min, *P < 0.05 for Ang vs sham. Family wise error rate was controlled using a modified Bonferroni procedure. **(B)** Changes after vehicle Ringers (200 nl) injection, averaged at 0–30 min.

Vehicle microinjection in the PVN had no effect on any parameter in either group (Figure 4B). PVN administration of RU28318 induced falls in MAP that were similar in sham and Ang II groups (−3.2 ± 1.1 mmHg and −2.4 ± 1.7 mmHg, respectively, P_*sham*_ > 0.5, Figure 4A). There was a decrease in heart rate in the Ang II group (−8 ± 3b/min) but not the sham (P_*sham*_ = 0.045, Figure 4A). RSNA also decreased from control by 12% in the Ang II rabbits (−0.8 ± 0.3nu) and this decrease was greater than the small fall observed in shams (P_*sham*_ = 0.049, Figure 4A).

**FIGURE 5:**
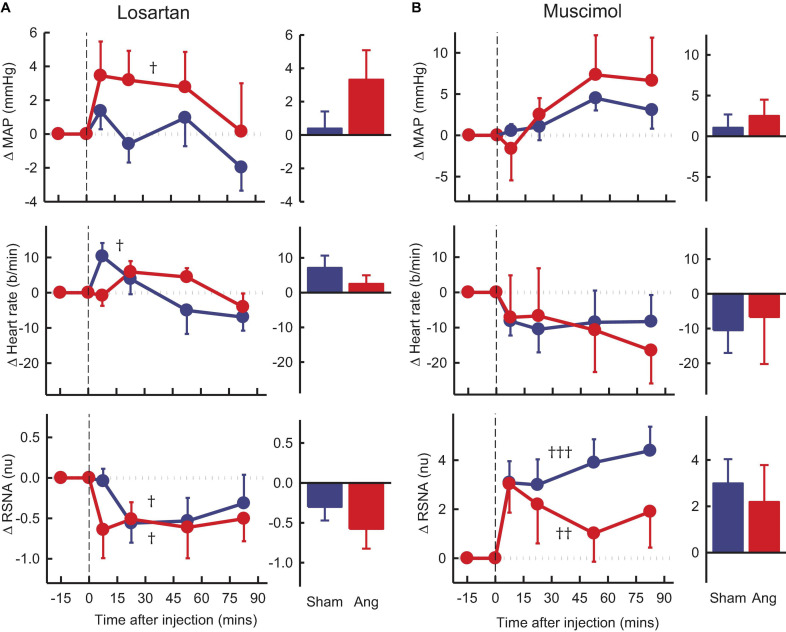
Average changes in mean arterial pressure (MAP), heart rate and renal sympathetic nerve activity (RSNA, normalized units) in rabbits treated with bilateral PVN microinjections of losartan and muscimol. **(A)** Line graphs are mean difference from control ± SED after losartan (5 nmol) microinjection shown at dashed vertical line in sham (blue circles, *n* = 7) and Ang II (red circles, *n* = 7) rabbits. ^†^*P* < 0.05, ^††^*P* < 0.01, and ^†††^*P* < 0.001 for change from control at 30–60 min. Bar graphs show average difference from control ± SED 0–30 min from injection. Family wise error rate was controlled using a modified Bonferroni procedure. **(B)** Changes after Muscimol (2 nmol) injection, averaged at 0–30 min.

Losartan, in the Ang II group, induced an average rise in MAP of +3.3 ± 1.8mmHg, P_*control*_ = 0.02) but a fall in RSNA of 12% (−0.6 ± 0.2nu, P_*control*_ = 0.03), neither of which was different to shams (Figure 5A). We observed no change in heart rate in the Ang II rabbits but a small increase in shams (Figure 5A).

Muscimol microinjection had no effect on MAP and heart rate in either group during the first 30 min. However, there was a rapid marked and sustained increase in RSNA which was similar in sham and Ang II groups (+3.6 ± 0.8nu, +31% and +2.6 ± 1.3nu, +37%, respectively, P_*control*_ < 0.01, Figure 5B).

### Effect of Antagonists and Agonist on Responses to Air Jet Stress

**FIGURE 6:**
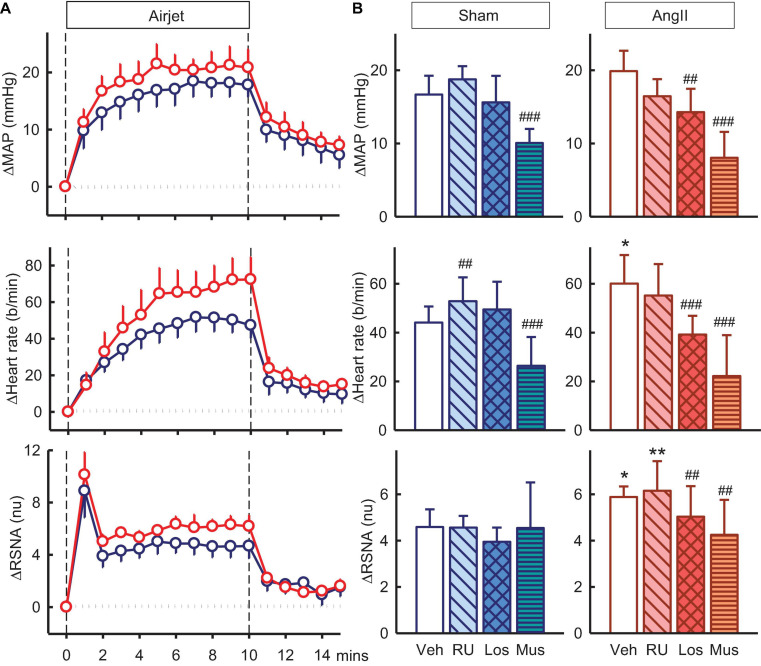
Effects of antagonists and agonist on mean arterial pressure (MAP), heart rate and total renal sympathetic nerve activity (RSNA, normalized units) responses to air jet stress. **(A)** Line graphs show time course of changes from control in vehicle-treated sham (blue circles) and Ang II (red circles) rabbits to air jet (vertical dashed lines). **(B)** Bar graphs are average change from control in sham **(left)** and Ang II **(right)** rabbits treated with vehicle (veh, white, *n* = 8–10), RU28318 (RU, *n* = 8–10, diagonal hatch), losartan (Los, *n* = 7, cross hatch), and muscimol (Mus, *n* = 5–6, horizontal hatch). Data are mean difference ± SED, *P < 0.05 and **P < 0.01 for Ang II vs sham; ^#^*P* < 0.05, ^##^*P* < 0.01, and ^###^*P* < 0.001 for antagonist or agonist vs vehicle.

Exposure to air jet stress induced increases in MAP, RSNA, and heart rate of +16.7 ± 2.6mmHg, +4.6 ± 0.8nu and +44 ± 7b/min in vehicle treated sham rabbits. The increases in RSNA and heart rate were greater in Ang II rabbits (28 and 36%, respectively, both P_*sham*_ < 0.05) but MAP was similar (Figure 6). The enhanced RSNA response in Ang II rabbits compared to sham was similar after RU28318 (P_*sham*_ = 0.008, Figure 6B). Losartan reduced the increases in MAP, heart rate and RSNA when compared to vehicle in the Ang II group (P_*veh*_ < 0.014, Figure 6B). However, muscimol in the PVN reduced both the MAP response in sham and Ang II groups compared to vehicle by 27–39% (+10.1 ± 1.9mmHg and +8.0 ± 3.5mmHg, both P_*veh*_ < 0.001), and the tachycardia by 28–48% (+26 ± 12b/min and +22 ± 17b/min, in sham and Ang II respectively, P_*veh*_ < 0.004, Figure 6B). Muscimol also markedly attenuated the sympatho-excitation to air jet but only in the Ang II group (+4.3 ± 1.5nu, −16%, P_*veh*_ = 0.015, Figure 6B).

### Effect of Antagonists and Agonist on Responses to Hypoxia

**FIGURE 7:**
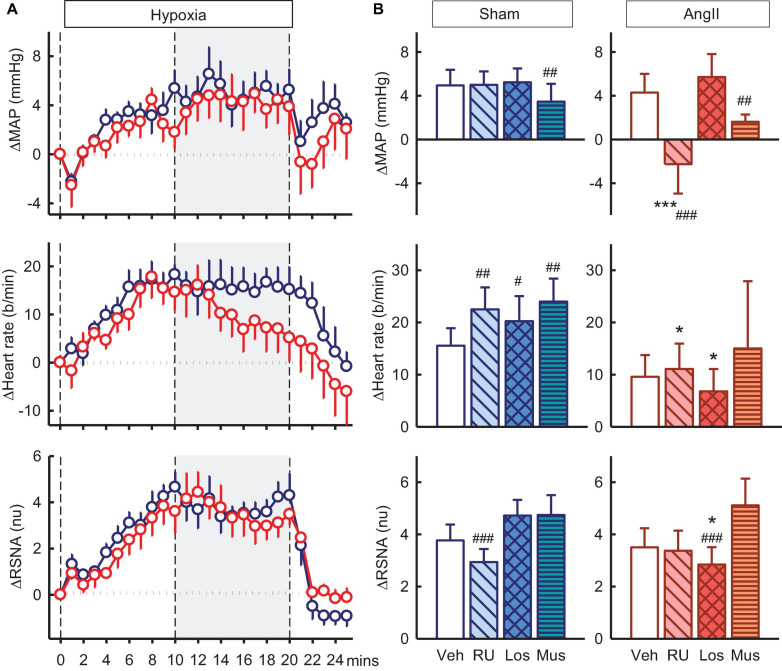
Effects of antagonists and agonist on mean arterial pressure (MAP), heart rate and total renal sympathetic nerve activity (RSNA, normalized units) responses to hypoxia. **(A)** Line graphs show time course of changes from control in vehicle-treated sham (blue circles) and Ang II (red circles) rabbits to hypoxia (vertical dashed lines). **(B)** Bar graphs are average change from control in sham **(left)** and Ang II **(right)** rabbits, measured during period shown by gray shading, treated with vehicle (veh, white, *n* = 8–10), RU28318 (RU, *n* = 8–10, diagonal hatch), losartan (Los, *n* = 7, cross hatch), and muscimol (Mus, *n* = 5–6, horizontal hatch). Data are mean difference ± SED, *P < 0.05 and ***P < 0.001 for Ang II vs sham; ^#^*P* < 0.05, ^##^*P* < 0.01, and ^###^*P* < 0.001 for antagonist or agonist vs vehicle.

Hypoxia also produced increases in MAP, RSNA, and heart rate of +4.9 ± 1.4mmHg, +3.8 ± 0.6nu, and +16 ± 3b/min in vehicle treated sham rabbits and increases in Ang II rabbits were similar (Figure 7A). RU28318 in the PVN abolished the rise in MAP in the Ang II group (−2.3 ± 2.7mmHg, P_*veh*_ < 0.001, P_*sham*_ < 0.001) and also similarly for heart rate (+11 ± 5b/min, P_*veh*_ = 0.04) (Figure 7B). The effect of losartan, by contrast, was to reduce the increase in RSNA in the Ang II rabbits compared to sham by 40% (+2.8 ± 0.7nu vs +4.7 ± 0.6nu, P_*sham*_ = 0.03) and vehicle by 26% (P_*veh*_ < 0.001, Figure 7B). Similar to the results for RU28318, the increase in heart rate was attenuated by losartan in Ang II rabbits compared to sham (+ 7 ± 4b/min vs + 20 ± 5b/min, P_*sham*_ = 0.03). Muscimol reduced the hypertensive response to hypoxia in both sham (+3.5 ± 1.6mmHg, −32%, P_*veh*_ = 0.009) and Ang II groups (+1.6 ± 0.7, −55%, P_*veh*_ = 0.005, Figure 7B) but had no effect on RSNA.

### Effect of Antagonists and Agonist on Baroreflexes

**FIGURE 8:**
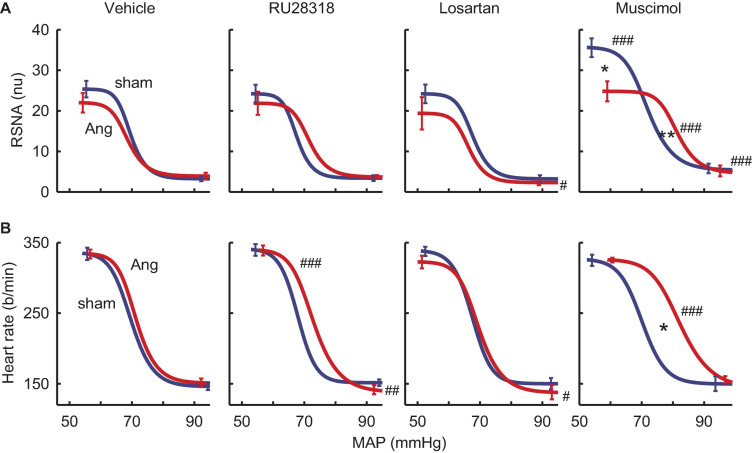
Effects of antagonists and agonist on baroreflexes. **(A)** MAP-renal sympathetic nerve activity (RSNA, normalized units) relationships after bilateral microinjection in the PVN of vehicle (*n* = 7–10), RU28318 (*n* = 7–10), losartan (*n* = 5–7), and muscimol (*n* = 4–6) in sham (blue) and Ang II (red) treated rabbits. **(B)** MAP-heart rate curves. Data are mean ± SEM. Family wise error rate was controlled using a modified Bonferroni procedure. *P < 0.05 and **P < 0.001 for Ang II vs sham; ^#^*P* < 0.05, ^##^*P* < 0.01, and ^###^P < 0.001 for antagonist or agonist vs vehicle.

RSNA baroreflexes were similar in sham and Ang II groups treated with vehicle. Microinjection of RU28318 and losartan did not alter this (Table 2). However, in the sham rabbits after muscimol treatment, the range and the maximum reflex RSNA response to lowering MAP were markedly greater than after vehicle (P_*veh*_ < 0.001) and compared to the Ang II group (P_*sham*_ = 0.009, Table 2, and Figure 8A). In the Ang II group, the curve after muscimol was shifted to the higher MAP compared to vehicle (P_*veh*_ < 0.001) but RSNA baroreflex sensitivity remained unchanged in all groups (Table 2 and Figure 8A). The heart rate baroreflex was also similar in sham and Ang II groups. We observed no effects of RU28318, losartan or muscimol in the sham group (Table 3). In the Ang II group treated with RU28318, the range of the heart rate baroreflex was 10% greater than in vehicle-treated rabbits mainly due to a similar reduction in the lower plateau (both P_*veh*_ < 0.01, Table 3, and Figure 8B). After muscimol in Ang II rabbits, the curve was shifted to the right compared to both vehicle (P_*veh*_ < 0.001) and to sham rabbits (P_*sham*_ = 0.028, Table 3, and Figure 8B). None of the treatments altered heart rate baroreflex sensitivity (Table 3).

**TABLE 2:** Renal sympathetic nerve baroreflex parameters.

	Sham										
Parameter	Vehicle	RU28318	P_*veh*_	Losartan	P_*veh*_	Muscimol	P_*veh*_				
Lower plateau (nu)	3.2 ± 0.6	3.4 ± 0.7	>0.5	3.2 ± 0.9	>0.5	5.4 ± 1.2	**<0.001**				
Range (nu)	22.1 ± 2	20.8 ± 2.2	>0.5	21 ± 2.3	0.055	30.3 ± 2.3	**<0.001**				
Upper plateau (nu)	25.4 ± 2.2	24.2 ± 2.4	>0.5	24.2 ± 3.1	0.19	35.7 ± 3	**<0.001**				
BP50 (mmHg)	69.5 ± 2.1	67.5 ± 1.8	>0.5	67.7 ± 3.3	>0.5	71.8 ± 2.1	>0.5				
Gain (nu/mmHg)	−2 ± 0.3	−2 ± 0.3	>0.5	−1.6 ± 0.2	0.34	−1.7 ± 0.3	0.17				
n	10	10		7		6					

	**Ang II**							**P_*sham*_**			
**Parameter**	**Vehicle**	**RU28318**	**P_*veh*_**	**Losartan**	**P_*veh*_**	**Muscimol**	**P_*veh*_**	**Veh**	**RU**	**Los**	**Mus**

Lower plateau (nu)	3.9 ± 0.8	3.6 ± 0.5	>0.5	2.3 ± 0.7	**<0.001**	4.6 ± 1.3	>0.5	>0.5	>0.5	>0.5	> 0.5
Range (nu)	18.1 ± 2.4	18.3 ± 2.9	>0.5	17.1 ± 4	>0.5	20.2 ± 2.5	>0.5	>0.5	>0.5	>0.5	**0.009**
Upper plateau (nu)	22 ± 1.7	21.9 ± 2.8	>0.5	19.4 ± 4.1	>0.5	24.8 ± 2.4	>0.5	>0.5	>0.5	>0.5	**0.009**
BP50 (mmHg)	68.6 ± 4.1	71.5 ± 4	>0.5	66.4 ± 2.3	>0.5	81.3 ± 8.8	**<0.001**	>0.5	>0.5	>0.5	0.21
Gain (nu/mmHg)	−1.4 ± 0.3	−1.4 ± 0.4	>0.5	−1.4 ± 0.4	>0.5	−1.3 ± 0.4	>0.5	>0.5	>0.5	>0.5	> 0.5
n	7	7		5		4					

*Data are mean ± SEM in sham (upper) and Ang II (lower) treated rabbits. P_*veh*_ for antagonist or agonist vs vehicle and P_*sham*_ for Ang II vs sham for each antagonist or agonist, shown in bold if *P* < 0.05. Veh, Vehicle; RU, RU28318; Los, Losartan; Mus, Muscimol; nu, normalized units.*

**TABLE 3:** Heart rate baroreflex parameters.

	Sham										
Parameter	Vehicle	RU28318	P_*veh*_	Losartan	P_*veh*_	Muscimol	P_*veh*_				
Lower plateau (b/min)	146 ± 5	151 ± 5	>0.5	150 ± 8	>0.5	150 ± 10	>0.5				
Range (b/min)	191 ± 9	190 ± 8	>0.5	189 ± 7	>0.5	178 ± 8	>0.5				
Upper plateau (b/min)	336 ± 5	341 ± 6	0.21	339 ± 5	>0.5	327 ± 4	>0.5				
BP50 (mmHg)	69.6 ± 1.7	67.9 ± 1.7	>0.5	67.5 ± 3.1	>0.5	70.1 ± 2.4	>0.5				
Gain (b/min/mmHg)	−12 ± 1.9	−14.2 ± 3	>0.5	−13.2 ± 1.6	>0.5	−10.6 ± 1.6	>0.5				
n	10	10		7		6					

	**Ang II**							**P_*sham*_**			
**Parameter**	**Vehicle**	**RU28318**	**P_*veh*_**	**Losartan**	**P_*veh*_**	**Muscimol**	**P_*veh*_**	**Veh**	**RU**	**Los**	**Mus**

Lower plateau (b/min)	151 ± 6	138 ± 6	**0.006**	137 ± 10	**0.013**	143 ± 5	0.31	>0.5	0.15	0.44	>0.5
Range (b/min)	184 ± 6	202 ± 7	**0.001**	186 ± 9	>0.5	183 ± 3	>0.5	>0.5	>0.5	>0.5	>0.5
Upper plateau (b/min)	335 ± 6	340 ± 7	0.18	323 ± 8	0.47	327 ± 6	>0.5	>0.5	>0.5	0.087	>0.5
BP50 (mmHg)	71.2 ± 4.3	72.8 ± 3.7	>0.5	69.5 ± 3.2	>0.5	82.2 ± 7.1	**<0.001**	>0.5	>0.5	>0.5	**0.028**
Gain (b/min/mmHg)	−12.9 ± 1.7	−11.5 ± 1.4	>0.5	−11.3 ± 1.8	>0.5	−8.3 ± 1	>0.5	>0.5	>0.5	>0.5	>0.5
n	8	8		6		5					

*Data are mean ± SEM in sham (upper) and Ang II (lower) treated rabbits. P_*veh*_ for antagonist or agonist vs vehicle and P_*sham*_ for Ang II vs sham for each antagonist or agonist, shown in bold if *P* < 0.05. Veh, Vehicle; RU, RU28318; Los, Losartan; Mus, Muscimol.*

## Discussion

In the present study we examined whether the hypertension induced by long term infusion of low dose Ang II involved activation of MR or AT_1_R in the PVN and modification of responses to baroreflexes, stress or chemoreceptor activation. We also assessed the overall contribution of the PVN to Ang II-induced changes in MAP using injections of the GABA_*A*_ agonist muscimol. The main findings (Table 4) were that blood pressure increased to a greater extent in the Ang II group compared to the sham group but RSNA was similar. The MR antagonist RU28318 administered directly to the PVN lowered blood pressure in both Ang II and sham treatment groups but had a greater effect on RSNA in the Ang II group. RU28318 also significantly attenuated the blood pressure response to chemoreceptor stimulation in this group but did not affect the response to air jet stress nor the RSNA baroreflex. In addition, RU28318 augmented the range of the heart rate baroreflex in Ang II rabbits. Together these data suggest that MR activation in the PVN contributes not only to sympatho-excitation following Ang II infusion, but also to the peripheral pressor response to hypoxia. By contrast, a lack of response to losartan suggests that, surprisingly, central AT_1_R play an inhibitory role in the maintenance of hypertension during a peripheral infusion and that they also have an excitatory action on the pressor response to air jet stress and the sympathetic response to hypoxia. Given that muscimol produced sympatho-excitation in both control and Ang II infused groups, these data suggest that the PVN is predominately sympatho-inhibitory, but this is independent of the Ang II hypertension. By contrast the PVN makes no contribution to the pressor or RSNA response to hypoxia. Interestingly, the effect of muscimol suggests that the PVN normally inhibits the range and upper plateau of the RSNA baroreflex, although this is not apparent in the Ang II group. Taken together, the PVN plays a relatively large role in modulating the SNS and the influence is very much driven by the type of stimulus and differs in the presence of Ang II hypertension.

**TABLE 4:** Summary of effects of stimuli on receptor signaling in the PVN.

Receptor	Stimulus	MAP	Heart rate	RSNA
MR	Angiotensin II	+	+	+
	Chemoreceptors	+	+	=
	Stress	=	=	=
	Baroreceptors		=	=
AT_1_R	Angiotensin II	−	=	+
	Chemoreceptors	=	+	+
	Stress	+	+	+
	Baroreceptors		=	=
GABA_*A*_R	Angiotensin II	=	=	−
	Chemoreceptors	+	−	=
	Stress	+	+	+
	Baroreceptors		=	−

*Effects on mean arterial pressure (MAP), heart rate and renal sympathetic nerve activity (RSNA) are shown as excitatory (+), inhibitory (−) or no effect ( = ). Signaling of mineralocorticoid receptors (MR), angiotensin type 1 receptors (AT_1_R) and GABA_*A*_ receptors (GABA_*A*_R).*

The PVN has many important roles in regulating body fluid homeostasis (Badoer, 1996), sodium and thirst sensing (Kapusta et al., 2013) as well as regulating endocrine and autonomic responses to stress (Benarroch, 2005; Oki et al., 2012). Indeed, PVN pre-sympathetic neurons have a significant role in longer term challenges such as hypoxia, pregnancy and hypertension in which they generate increased sympathetic vasomotor activity (Dampney et al., 2018). The contribution of the PVN to tonic sympathetic drive has also been clearly demonstrated in heart failure (Patel and Zhang, 1996), genetic hypertension (Akine et al., 2003; Li and Pan, 2007), aldosterone induced hypertension (Xue et al., 2011) and polycystic kidney induced hypertension (Underwood et al., 2019). PVN directed control of peripheral responses is achieved through (i) inputs ranging from peripheral receptors, higher brain regions including the amygdala and cortex, ascending inputs from the medulla, as well as circulating factors such as Ang II and other hormones and (ii) major outflows to the spinal preganglionic sympathetic neurons, the RVLM and dorso-vagal complex (Dampney et al., 2018). Further, Ang II hypertension is also dependent on a degree of plasticity of NMDA receptor GluN1 subunit expression (Glass et al., 2015). Neurons within the parvocellular region of the PVN are an important component of the central neurocircuitry regulating efferent SNA by influencing the basal level of activity and the patterns of differential bursts relevant for particular nerves innervating different target organs (Kenney et al., 2003). Injection of the sympatho-excitatory cholinergic agonist carbachol into the PVN of conscious rats increases blood pressure and constricts renal, superior mesenteric and hindquarters vascular beds (Bachelard et al., 1994). Most studies have shown a relatively small contribution of the PVN in normal animals based on small increases or decreases in blood pressure with muscimol injections (Ng et al., 2004; Stocker et al., 2004; Freeman and Brooks, 2007; Underwood et al., 2019). This contrasts with situations that change plasma osmolarity such as water deprivation in which the PVN plays a major sympatho-excitatory role (Stocker et al., 2004, 2005; Freeman and Brooks, 2007). By contrast the PVN is sympatho-inhibitory during infusions of hypertonic saline (Badoer et al., 2003; Ng et al., 2004).

Our interest, however, was in the role of the PVN and in particular the contribution of MR and AT_1_R to Ang II induced hypertension. Previous studies have shown that chronic administration of the AT_1_R antagonist losartan directly into the PVN attenuates hypertension in a sleep apnea model (da Silva et al., 2011). In the present study we found that blocking MR lowered blood pressure in both Ang II and sham rabbits, and moreover that MR blockade had a greater effect on RSNA and heart rate in the Ang II group. Thus, MR in the PVN did not appear to contribute selectively to the onset of hypertension, and instead MR played an important role in renal sympathetic excitation. By contrast losartan administered directly into the PVN increased blood pressure only in the Ang II group, and lowered RSNA in both sham and Ang II groups. This is quite a different pattern of response when compared to that for the MR antagonist RU28318 and suggests that there is chronic activation of AT_1_R in the PVN that opposes the development of hypertension.

Moreover, our findings contrast with those of Chen and colleagues who showed that reducing MR or AT_1_R gene expression in the PVN prevents Ang II hypertension in rats (Chen A. et al., 2014). This group extended their findings to show that an infusion of small interfering RNA against MR or AT_1_R into the SFO prevented most of the Ang II-induced hypertension and that the mechanism involving Ang II induction of reactive oxygen species in the PVN required activation of MR signaling in the SFO (Wang et al., 2016). However, these effects were observed with a much greater dose of Ang II infused for 2 weeks (500 ng/kg/min) which produced a greater hypertensive response (∼50% increase in blood pressure), compared to the low dose of Ang II used in the present study (24 ng/kg/min) and the modest 20% hypertension. This dose was chosen based on our previous study on the effects of 3 months infusion with Ang II that produced hypertension, sympathetic-excitation and alteration to sympathetic reflexes and responses (Moretti et al., 2012).

We have previously shown both acute infusions of a higher dose of Ang II (50 ng/kg/min) over a few hours, 3 days and 2 weeks have produced sustained activation of a range of central nuclei including circumventricular organs such as the OVLT, and SFO as well as the PVN and SON (Davern and Head, 2007). Thus, it was somewhat unexpected that the antagonists microinjected into the PVN had such small effects. These data are contrary to the view expressed by Leenan that MR and AT_1_R are involved at least in the acute (2 week) phase of Ang II hypertension (Leenen, 2014). However, we would also suggest that the degree of involvement of the PVN may well depend on the severity of the stimulus as well as the phase of Ang II induced hypertension. Moreover, there may be differences in the role of brain regions between the early phase at 2 weeks versus more prolonged treatment (3 months). It is also possible that species differences contribute to the disparity in the results, as the studies by Chen A. et al. (2014) and Wang et al. (2016) were conducted in rats. It should also be considered that while Ang II administration can promote aldosterone synthesis, the ligand for the MR in the regions of the brain under consideration here may not always be aldosterone. Cortisol circulates at substantially higher levels than for aldosterone, and 11bHSD2 is variable and lower than for the kidney; the MR in the PVN may thus respond to cortisol at least at some points. Cell stress (oxidative) can promote cortisol agonist activity in non-epithelial tissues and the effect of RU28318 in the brain may be due in part to blockade of cortisol bound MR in these regions.

We have previously reported that 12 weeks of Ang II infusion enhanced the sympathetic response to air jet stress and hypoxia (Moretti et al., 2012) and in the current study, we have examined the contribution of MR and AT_1_R in the PVN to these responses. Although the PVN is more known for its contribution to the neuroendocrine response to stress rather than the sympatho-excitation and cardiovascular response (Stotz-Potter et al., 1996), hypoxia can activate neurons in the NTS projecting to the PVN (King et al., 2012). Further, losartan given ICV attenuates the pressor response and the FosB response in the PVN to chronic intermittent hypoxia (Knight et al., 2013). Interestingly in our study, neither MR nor AT_1_R contributed to the RSNA response to air jet stress but losartan reduced the pressor and tachycardic response. Losartan slightly attenuated the RSNA response to hypoxia while the MR antagonist abolished the pressor response to hypoxia in the Ang II group but had no effect in the sham animals. Thus, we can conclude there is only a relatively minor contribution of MR or the AT_1_R to these sympathetic stimuli.

A strength of the current study was that each pharmacological blockade was performed in the same rabbits on alternate days which enabled a within animal comparison. Importantly we used conscious rabbits which avoided the issues often seen with anesthetized preparations (Mayorov et al., 2001). The size of the rabbit brain is also a distinct advantage as we can inject into discrete nuclei with a great deal of accuracy (see Figure 2) with a high success rate (19 out of 23). Importantly, based on previous studies injecting into the dorsomedial or ventromedial hypothalamus (Lim et al., 2016), we can confine injections to within 0.5 mm and distinguish between effects in adjacent nuclei. In the present study we used a relatively low dose of Ang II due to difficulties of using high doses suppressing RSNA presumably through baroreflex mechanisms as previously reported using this model (Moretti et al., 2012). Our aim was to use what is considered a slow pressor level of Ang II stimulation and allow several weeks or months for the sympatho-excitation to manifest. We did notice that in the current study that there was a rise in blood pressure particularly during the latter phase in the sham animals. Moreover, RSNA reported for the sham group was equivalent to the Ang II group, which is in contrast to previous studies from this group. Comparing the absolute values, the levels of RSNA in the sham group are nearly double that of sham rabbits in our previous studies (Moretti et al., 2012). Perhaps the limitation of our current design was that a subtle dose of Ang II and the positioning of a guide cannula within the hypothalamus may have been responsible for minimizing the differences between groups by adding another level of stress. Indeed, the RSNA response to air jet stress was quite large and almost double that observed previously (Moretti et al., 2012).

In conclusion, we suggest that after 3 months of moderate Ang II induced hypertension, the activation of sympathetic activity to the kidney is mediated principally through MR activation in the PVN rather than activation of AT_1_R but the hypertension is independent of both. The sympatho-excitatory effects of muscimol in both groups suggests that overall, the PVN is providing a tonic sympatho-inhibitory influence but this is independent of Ang II induced hypertension.

## Disclosure

GH has received research support from Boehringer Ingelheim for studies unrelated to the current study.

## Data Availability Statement

The raw data supporting the conclusions of this article will be made available by the authors, without undue reservation.

## Ethics Statement

The animal study was reviewed and approved by Alfred Medical Research Education Precinct Animal Ethics Committee.

## Author Contributions

GH and MY designed the study. All authors performed the study including experimental data collection and analysis, preparation, and writing and editing of the manuscript.

## Conflict of Interest

The authors declare that the research was conducted in the absence of any commercial or financial relationships that could be construed as a potential conflict of interest.
